# Functional changes in adipose tissue in a randomised controlled trial of physical activity

**DOI:** 10.1186/1476-511X-11-80

**Published:** 2012-06-21

**Authors:** Per Sjögren, Justo Sierra-Johnson, Lena V Kallings, Tommy Cederholm, Maria Kolak, Mats Halldin, Kerstin Brismar, Ulf de Faire, Mai-Lis Hellénius, Rachel M Fisher

**Affiliations:** 1 Atherosclerosis Research Unit,, Department of Medicine, (Solna) Karolinska Institutet,, Stockholm, Sweden; 2 Clinical Nutrition and Metabolism, Department of Public Health and Caring Sciences, Uppsala University, Uppsala, Sweden; 3 Cardiovascular Division, Mayo Clinic, Rochester, MN, USA; 4 Department of Sport and Health Sciences, Swedish School of Sport and Health Sciences, Stockholm, Sweden; 5 Unit of Cardiology, Department of Medicine, (Solna) Karolinska Institutet, Stockholm, Sweden; 6 Institute of Environmental Medicine, Division of Cardiovascular Epidemiology, Karolinska Institutet, Stockholm, Sweden; 7 Rolf Luft Research Center for Diabetes and Endocrinology, , Department of Molecular Medicine and Surgery, Karolinska Institutet, , Stockholm, Sweden

**Keywords:** Adipose tissue, Physical activity, Fatty acid composition, Gene expression

## Abstract

**Background:**

A sedentary lifestyle predisposes to cardiometabolic diseases. Lifestyle changes such as increased physical activity improve a range of cardiometabolic risk factors. The objective of this study was to examine whether functional changes in adipose tissue were related to these improvements.

**Methods:**

Seventy-three sedentary, overweight (mean BMI 29.9 ± 3.2 kg/m^2^) and abdominally obese, but otherwise healthy men and women (67.6 ± 0.5 years) from a randomised controlled trial of physical activity on prescription over a 6-month period were included (control n = 43, intervention n = 30). Detailed examinations were carried out at baseline and at follow-up, including fasting blood samples, a comprehensive questionnaire and subcutaneous adipose tissue biopsies for fatty acid composition analysis (n = 73) and quantification of mRNA expression levels of 13 candidate genes (n = 51), including adiponectin, leptin and inflammatory cytokines.

**Results:**

At follow-up, the intervention group had a greater increase in exercise time (+137 min/week) and a greater decrease in body fat mass (−1.5 kg) compared to the control subjects (changes of 0 min/week and −0.5 kg respectively). Circulating concentrations of adiponectin were unchanged, but those of leptin decreased significantly more in the intervention group (−1.8 vs −1.1 ng/mL for intervention vs control, *P* < 0.05). The w6-polyunsaturated fatty acid content, in particular linoleic acid (18:2w6), of adipose tissue increased significantly more in the intervention group, but the magnitude of the change was small (+0.17 vs +0.02 percentage points for intervention vs control, *P* < 0.05). Surprisingly leptin mRNA levels in adipose tissue increased in the intervention group (+107% intervention vs −20% control, *P* < 0.05), but changes in expression of the remaining genes did not differ between the groups.

**Conclusions:**

After a 6-month period of increased physical activity in overweight elderly individuals, circulating leptin concentrations decreased despite increased levels of leptin mRNA in adipose tissue. Otherwise, only minor changes occurred in adipose tissue, although several improvements in metabolic parameters accompanied the modest increase in physical activity.

## Introduction

The number of obese individuals worldwide has increased dramatically during the last couple of decades. Obesity strongly predisposes to cardiometabolic diseases and hence, a vast number of people are characterized with a poor health prognosis.

A major contributor to the obesity epidemic in modern societies is a sedentary lifestyle and low levels of daily physical activity have a negative effect on many physiological pathways [1]. Increased physical activity, as induced for example by an individualised written prescription, has been shown to improve a spectrum of clinical risk markers, and hence cardiometabolic risk [2-4]. Furthermore, increasing the degree of physical activity, as one part of a healthier lifestyle, has shown even greater efficiency than pharmacotherapy in preventing the onset of type 2 diabetes [5]. Such data clearly support the promotion of physical activity as a key factor in the battle of primary prevention. Metabolic pathways that are affected by increased physical activity include weight regulation, glucose and lipid handling capacities, hemodynamics, hormonal balance and inflammatory state [6-8]. All of these variables are recognised components of the unfavourable metabolic state called the metabolic syndrome. However, the mechanisms at the molecular level by which changes in physical activity lead to improvements in metabolic parameters have not been fully elucidated. One possibility is that changes in adipose tissue metabolism and function are involved, either directly or indirectly, in these metabolic improvements.

Obesity is tightly related to insulin resistance and adipose tissue is an important endocrine organ that produces adipokines that can affect metabolic and inflammatory pathways, predominantly in an autocrine/paracrine fashion, but also systemically [9-11]. Relationships between many of these adipokines (such as adiponectin, leptin, IL-6 and TNFα) and insulin resistance have been described [12,13]. Obesity and insulin resistance are strongly associated with local inflammation and macrophage accumulation within adipose tissue, and a direct relationship between adipose tissue inflammation and insulin resistance has been proposed [12,14,15]. However, more recently a “house keeping” role of adipose tissue macrophages in the regulation of adipocyte lipolysis has been suggested [16], indicating the complexity of the relationship between local inflammation, adipose tissue function and obesity/insulin resistance.

Another important aspect of adipose tissue metabolism in relation to components of the metabolic syndrome is the fatty acid composition of stored triglycerides. A higher content of saturated fatty acids has been described in adipose tissue from obese compared to overweight individuals [17], while a diet rich in saturated fatty acids promoted expression in adipose tissue of genes involved in inflammation [18]. Estimates of the activity of stearoyl CoA desaturase (SCD) in adipose tissue have been positively correlated to insulin resistance [19] and obesity [20], possibly suggesting an increased desaturation of adipose tissue fatty acids by SCD in response to (and to cope with) an unfavourable increase in saturated fatty acids.

Therefore in this randomized controlled trial in overweight individuals we investigated whether increases in physical activity over a 6-month period induced changes in subcutaneous adipose tissue as assessed by changes in i) circulating adiponectin and leptin concentrations, ii) adipose tissue fatty acid composition, and iii) expression in adipose tissue of genes encoding key proteins.

## Materials and methods

### Study subjects and study design

Study design and study participants have been described in detail elsewhere [3]. In brief, 101 overweight (BMI ≥25 kg/m^2^ and <40 kg/m^2^), centrally obese (waist circumference ≥102 cm in men and ≥88 in women) [21], physically inactive, but otherwise healthy individuals (67–68 years) were recruited from a Stockholm county-cohort [22] to participate in a life-style intervention study over 6 months. Recruitment took place between January and June 2006. The present study of adipose tissue was completed at the 6 month follow-up. The study was performed at Karolinska University Hospital, Huddinge. Participants were randomized in parallel fashion to either a control group (n = 54) or to an exercise intervention group (n = 47) with a baseline and a 6 month follow-up. Calendar days were randomised as either control or intervention days to prevent discussions between subjects in the different groups. Study participants and staff, apart from those staff directly involved in the intervention, were blinded to intervention status. Blood samples were taken after an overnight fast. In 73 of the subjects (29 men, 44 women), it was possible to take a subcutaneous abdominal adipose tissue biopsy approximately 5 cm lateral to the umbilicus at baseline and again after 6 months under local anaesthesia by needle biopsy. However, the collection of biopsies was not evenly distributed across the two groups: n = 43 control, n = 30 intervention. The fatty acid composition of these biopsies was determined (see below). In a subset of biopsies (n = 51, 21 men, 30 women), in which there was sufficient material for RNA extraction, gene expression analysis was performed. These individuals from whom adipose tissue biopsies were taken (n = 73 for fatty acid composition and n = 51 for gene expression analysis) represent the current study population. The primary outcomes were differences between the intervention and control groups in changes in adipose tissue metabolism as assessed by circulating adiponectin and leptin concentrations and adipose tissue fatty acid composition and gene expression.

The degree of physical activity was assessed by pedometry and by an activity diary over seven consecutive days. Information on exercise time was obtained, and physical activity of at least moderate intensity was assessed as described [3]. The intervention group received patient-centred counselling and individualized written prescription of physical activity, as described [3]. In brief, the intervention aimed to achieve a daily physical activity level of at least 30 min of moderate intensity and included both aerobic and strength training. Participants were also encouraged to reduce their time spent in sedentary behaviour. No dietary recommendations were given. Due to ethical considerations, the control group received usual care including general information about the importance of physical activity for health. No side effects were reported during the study. All participants completed a food frequency questionnaire (28 questions) covering the most frequently consumed foods and beverages. Dietary improvements over the 6-month period were considered to have been made if consumption of vegetables, fruit or seafood increased, or if consumption of candy, buns, snacks, high fat cheese, pizza or soda decreased. If individuals improved their consumption of at least three of these food groups they were categorised as having favourably changed their diet.

The Ethics Committee of Karolinska Institutet approved the study and all subjects gave informed consent to participate.

### Assessment of anthropometry and cardiometabolic risk factors

Anthropometric measurements, body fat mass (bioelectrical bioimpedance) and blood pressure were assessed as described [3]. Glucose, glycosylated haemoglobin (HbA1c), cholesterol, HDL-cholesterol, triaclyglycerol, apolipoprotein A1 (apo A1), apolipoprotein B (apo B) and C-reactive protein (CRP) were analysed by accredited methods in the clinical chemistry laboratory at the Karolinska University Hospital, Huddinge. Insulin was quantified by ELISA (Dako Sweden) and adiponectin and leptin by radioimmunoassay (Linco Research, St Charles, Missouri, USA and Millipore, Billerica, MA, USA respectively). Homeostasis model assessment (HOMA) index was calculated as the product of fasting insulin and glucose concentrations divided by 22.5.

### Adipose tissue fatty acid composition analysis

Fatty acids in adipose tissue-triacylglycerols were separated by gas liquid chromatography as previously described [20] and expressed as the relative molar percentage of the sum of the fatty acids analysed. The fatty acids quantified were 14:0, 15:0, 16:0, 16:1w7, 17:0, 18:0, 18:1, 18:2w6, 18:3w6, 18:3w3, 20:3w6, 20:4w6, 20:5w3, 22:4w6, 22:5w3 and 22:6w3.

### RNA isolation from adipose tissue and cDNA synthesis

Following collection of subcutaneous adipose tissue biopsies, the samples were rinsed immediately in 0.9% NaCl to remove excess blood and stored in RNAlater (Qiagen) at −80 °C until analyzed. RNA was extracted from approximately 150 mg tissue: homogenization in phenol-containing TRIzol (Invitrogen), DNaseI treatment and spin column purification (RNeasy, Qiagen). RNA concentrations were determined using a NanoDrop spectrophotometer (Thermo) and the quality analyzed with an Agilent Bioanalyzer 2100 (Agilent Technologies). Isolated RNA was stored at −80 °C until cDNA synthesis. A total of 1 μg total RNA was used for cDNA synthesis using oligo-(dT)12-15 primers.

### Quantification of gene expression

The mRNA expression of specific genes was quantified by real time PCR using the ABI 7000 Sequence Detection System instrument and software (Applied Biosystems). In each reaction, cDNA that had been synthesized from 15 ng of total RNA was mixed with TaqMan Universal PCR Master Mix (Applied Biosystems) and a gene-specific primer and probe mixture (pre-developed TaqMan Gene Expression Assays, Applied Biosystems) in a final volume of 25 μl. The genes analysed and the corresponding assays used were: 11β-hydroxysteroid dehydrogenase type 1 (11βHSD1), Hs00194153_m1; adiponectin, Hs00605917_m1; monocyte chemoattractant protein 1 (CCL2), Hs00234140_m1; CD36, Hs00169627_m1; CD68, Hs00154355_m1; cannabinoid receptor 1 (CNR1), Hs00275634_m1; cannabinoid receptor 2 (CNR2), Hs00361490_m1; interleukin 6 (IL-6), Hs00174131_m1; leptin, Hs00174877_m1; lipoprotein lipase (LPL), Hs00173425_m1; peroxisome proliferator activated receptor gamma (PPARγ), Hs00234592_m1; stearoyl-CoA desaturase (SCD); Hs001682761_m1; tumour necrosis factor α (TNFα), Hs00174128_m1; and ribosomal protein large P0 (RPLP0), Hs99999902_m1. All samples were run in duplicate. Relative expression levels were determined using a 5-point serially diluted standard curve, generated from cDNA from human adipose tissue. Gene expression was expressed in arbitrary units and normalized relative to the housekeeping gene RPLP0 to compensate for differences in cDNA loading. Levels of RPLP0 mRNA were comparable between all subjects in the study.

### Statistical analysis

Data were summarized by calculating means and standard deviations (SD) or median and interquartile ranges (IQR) depending on normality of quantitative variables. Changes from baseline to follow-up were determined by paired *t*-test if normally distributed or by Wilcoxon matched-pair signed-rank test if skewed. ANCOVA, in which baseline values were taken into consideration, was used to analyse differences between the groups over the 6-month period. To investigate the ability of changes in physical activity, anthropometric measures and HbA1c (the parameters that showed significantly different responses between control and intervention groups) to predict changes in selected markers of adipose tissue metabolism, linear regression was applied. Skewed variables were normalised prior to analysis. All analyses were performed with the use of STATA statistical package (Intercooled STATA 11.0 for Windows; Stata Corp, College Station, TX), and significance was set at *P* < 0.05.

## Results

### Changes in physical activity, anthropometric measurements and metabolic status

Characteristics of participants in the control and intervention groups at baseline and their changes at 6 months are presented in Table 1. At baseline the control group had significantly higher BMI, waist circumference, body fat mass, insulin and HOMA index than the intervention group. Following the 6-month period, changes in physical activity, anthropometric measurements and diet differed significantly between the groups. The intervention group demonstrated greater increases in exercise time, greater decreases in weight, BMI and body fat mass, and greater dietary improvements compared to the control subjects. HbA1c was lowered in the intervention compared to the control group, but there were no significant differences between the groups with regard to changes in blood pressure or circulating concentrations of insulin, lipids or CRP. Circulating concentrations of adiponectin were unchanged over the 6-month period, with no differences between the control and intervention groups. However, concentrations of leptin decreased in both the control and intervention groups, although the decrease was greater in the intervention group (−1.8 vs −1.1 ng/mL for intervention vs control, *P* = 0.01).

**Table 1:** Baseline characteristics and follow-up changes of selected variables

	**Baseline**^**a**^		**Change**^**b**^		**Between group diff**
	**Control n = 43**	**Intervention n = 30**	**Control**	**Intervention**	**P**^**ANCOVA**^
Age (yr)	67.5 (0.5)	67.6 (0.5)	-	-	
Female (%)	58	63	-	-	
Exercise time (min/w)	120 (5, 205)	135 (40, 215)	0 (-105, 240)	+137 (0, 490)^**^	0.03
Steps per day	5200 (2730)	5900 (2800)	+719 (2490)	+1190 (3270)	0.28
Dietary improvements (%)^c^	-	-	12	49	0.001
Weight (kg)	89 (81, 95)	82 (73, 97)	-0.1 (-1.1, 0.9)	-1.8 (-3.9, 0.3)^**^	0.02
BMI (kg/m^2^)	30.3 (28.6, 31.8)^††^	27.5 (26.6, 30.8)	-0.04 (-0.47, 0.30)	-0.68 (-1.21, 0.11)^**^	0.04
Waist circumference (cm)	107 (8)^†^	103 (10)	-1.3 (3.1)^**^	-2.6 (4.0)^**^	0.12
Body fat mass (kg)	31.4 (28.1, 38.7)^†^	29.8 (24.7, 31.6)	-0.5 (-1.9, 0.4)^*^	-1.5 (-3.6, -0.4)^**^	0.04
Systolic blood pressure (mmHg)	144 (17)	136 (16)	-5 (11)^*^	-0 (15)	0.61
Diastolic blood pressure (mmHg)	81 (9)	78 (10)	-1 (8)	-1 (9)	0.61
Insulin (uU/mL)	10.9 (6.8, 14.1)^†^	8.2 (6.4, 10.9)	-0.85 (-2.98, 0.89)	-0.90 (-2.97, 0.46)^*^	0.16
Glucose (mmol/L)	5.4 (4.9, 5.7)	5.3 (4.9, 5.5)	-0.2 (-0.4, 0.1)^**^	-0.1 (-0.3, 0.1)	0.79
HOMA	2.6 (1.7, 3.6)^†^	1.9 (1.7, 2.3)	-0.2 (-0.9, 0.3)	-0.2 (-0.7, 0.1)	0.19
HbA1c (%)	4.8 (4.6, 5.0)	4.9 (4.7, 5.2)	0.1 (0.0, 0.3)^**^	-0.1 (-0.2, 0.1)	0.0003
Triacylglycerol (mmol/L)	1.2 (1.0, 1.6)	1.1 (1.0, 1.5)	-0.0 (-0.3, 0.2)	-0.1 (-0.3, 0.1)^*^	0.23
Cholesterol (mmol/L)	5.6 (0.9)	5.9 (1.0)	0.0 (0.6)	-0.3 (1.0)	0.14
HDL (mmol/L)	1.7 (1.4, 1.9)	1.7 (1.5, 1.9)	-0.1 (-0.1, 0.1)	+0.0 (-0.2, 0.2)	0.57
ApoB/A1	0.73 (0.14)	0.78 (0.20)	-0.04 (0.11)^*^	-0.08 (0.15)^**^	0.27
C-reactive protein (mg/L)	1.9 (1.1, 4.0)	1.7 (0.8, 3.4)	+0.2 (-0.4, 1.5)	+0.1 (-0.7, 0.6)	0.20
Adiponectin (mg/L)	16 (10, 21)	20 (14, 24)	-0.1 (-1.5, 1.3)	+0.3 (-1.0, 1.3)	0.21
Leptin (ng/mL)	22 (13, 36)	15 (13, 23)	-1.1 (-5.0, 0.5)	-1.8 (-8.8, 0.3) ^**^	0.011

Values are mean (SD) or median (IQR) and changes denote measure_(follow-up)_ – measure_(baseline)_.

Total number of individuals analysed varies slightly due to technical reasons.

^a^ Significant differences between groups at baseline: †*P* < 0.05, ††*P* < 0.01.

^b^ Significant within-group changes: *P < 0.05,**P < 0.01.

^c^ Defined as changes (according to self-reported frequencies) in the consumption of at least three of the following dietary variables: vegetables, fruit, seafood, candy, buns, snacks, high fat cheese, pizza and soda.

HOMA, homeostasis model assessment of insulin resistance; HDL, high density lipoprotein; ApoB/A1, ratio between apolipoproteins B and A1.

### Changes in adipose tissue fatty acid composition

The adipose tissue fatty acid profiles of the control and intervention groups at baseline and their changes at 6 months are presented in Table 2. After 6 months the w6-polyunsaturated fatty acid content of adipose tissue in the intervention group increased significantly more than in the controls (*P* = 0.04), which appeared to be explained by changes in linoleic acid, 18:2w6 (+0.17 vs +0.02 percentage points for intervention vs control, *P* = 0.01). There were no significant differences between the groups in changes of any other fatty acid or of an estimate of SCD activity (the 16:1/16:0 ratio).

**Table 2:** Baseline values and follow-up changes of fatty acids in subcutaneous adipose tissue

	**Baseline**^**a**^		**Change**^**b**^		**P**^**ANCOVA**^
	**Control n = 43**	**Intervention n = 30**	**Control**	**Intervention**	
**Grouped fatty acids**					
Saturated	30.4 (2.9)	31.0 (3.7)	-0.3 (1.3)	-0.2 (1.4)	0.48
Monounsaturated	57.0 (2.7)	56.5 (3.7)	+0.3 (1.3)	+0.1 (1.5)	0.33
Polyunsaturated w6	10.6 (9.4, 11.9)	10.4 (9.7, 11.3)	+0.1 (-0.2, 0.3)	+0.2 (-0.0, 0.5)^**^	0.04
Polyunsaturated w3	2.0 (0.4)	2.0 (0.5)	+0.0 (0.2)	-0.0 (0.1)	0.24
**Individual fatty acids**					
14:0	3.3 (0.5)	3.4 (0.7)	-0.10 (0.20)^**^	-0.11 (0.24)^*^	0.90
15:0	0.33 (0.06)	0.35 (0.08)	+0.01 (0.02)^**^	-0.00 (0.02)	0.12
16:0	23.1 (2.1)	23.1 (2.6)	-0.15 (0.89)	+0.01 (0.92)	0.45
16:1w7	6.4 (1.8)	5.7 (1.4)	+0.16 (0.68)	+0.04 (0.62)	0.28
17:0	0.27 (0.24, 0.30)	0.28 (0.25, 0.33)	+0.02 (-0.01, 0.06)^**^	+0.00 (-0.04, 0.06)	0.26
18:0	3.4 (0.7)^†^	3.9 (0.9)	-0.08 (0.34)	-0.08 (0.38)	0.50
18:1	50.6 (1.9)	50.7 (2.7)	+0.15 (0.88)	+0.06 (1.13)	0.78
18:2w6	9.6 (8.4, 11.0)	9.4 (8.7, 10.4)	+0.02 (-0.14, 0.22)	+0.17 (0.03, 0.39)^**^	0.012
18:3w6	0.10 (0.09, 0.11)	0.10 (0.09. 0.11)	+0.01 (0.00, 0.01)^**^	+0.00 (0.00, 0.01)	0.26
18:3w3	1.08 (0.24)	1.04 (0.25)	-0.02 (0.09)	-0.03 (0.10)	0.47
20:3w6	0.21 (0.17, 0.24)	0.20 (0.16, 0.24)	-0.01 (-0.03, 0.01)^**^	-0.01 (-0.02, 0.01)	0.72
20:4w6	0.43 (0.09)	0.40 (0.10)	+0.01 (0.05)	+0.01 (0.06)	0.52
20:5w3	0.16 (0.14, 0.19)	0.16 (0.14, 0.19)	+0.02 (-0.01, 0.05)^**^	-0.00 (-0.02, 0.03)	0.079
22:4w6	0.13 (0.12, 0.17)	0.14 (0.11, 0.17)	-0.00 (-0.01, 0.01)	-0.00 (-0.01, 0.01)	0.25
22:5w3	0.39 (0.10)	0.41 (0.14)	+0.00 (0.03)	-0.01 (0.05)	0.63
22:6w3	0.39 (0.13)	0.41 (0.19)	+0.01 (0.05)	+0.00 (0.05)	0.82
SCD-index (16:1/16:0)	0.28 (0.08)	0.26 (0.08)	+0.01 (0.04)	-0.00 (0.04)	0.13

Values are mean ± SD or median (IQR) and changes denote measure_(follow-up)_ – measure_(baseline)_. Fatty acids are presented as relative percentage of fatty acids analysed. Saturated, sum of 14:0, 15:0, 16:0, 17:0 and 18:0; Monounsaturated, sum of 16:1 and 18:1 Polyunsaturated w6, sum of 18:2, 18:3, 20:3, 20:4 and 22:4 (all w6); and Polyunsaturated w3, sum of 18:3, 20:5, 22:5 and 22:6 (all w3).

^a^ Significant differences between groups at baseline: †*P* < 0.05.

^b^ Significant within-group changes: *P < 0.05,**P < 0.01.

### Changes in adipose tissue gene expression

Characteristics of the subset of participants from whom adipose tissue gene expression data were available are presented in Additional file 1: Table S1. Changes in subcutaneous adipose tissue gene expression from baseline to follow-up in control and intervention groups are shown in Table 3. After 6-months there was a significant difference in the change in adipose tissue leptin mRNA levels between the groups, with an increase in the intervention group (+107% intervention vs −20% control, *P* < 0.02), a finding that was in contrast to the decreases in circulating leptin concentrations observed in both groups. There were no significant differences between groups for changes in adipose tissue expression levels of the remaining genes.

**Table 3:** Changes from baseline to follow-up in subcutaneous adipose tissue gene expression

	**Median percent change (IQR)**		**P**^**ANCOVA**^
	**Control**^**a**^ **n = 30**	**Intervention**^**a**^ **n = 21**	
CD68	0 (-39, 59)	-16 (-32, 20)	0.17
CCL2	-11 (-25, 66)	-17 (-39, 11)	0.11
IL-6	-31 (-56, 7)^**^	-16 (-62, 30)	0.87
TNFα	-11 (-57, 38)	-16 (-45, 42)	0.97
Adiponectin	-34 (-66, 64)	+38 (-42, 59)	0.35
Leptin	-20 (-54, 195)	+107 (-15, 428)	0.019
CD36	-8 (-16, 9)	-10 (-47, 18)	0.55
LPL	-3 (35, 26)	-24 (-43, 12)	0.44
PPARγ	-9 (-31, 21)	-11 (-28, 26)	0.30
11βHSD1	-4 (-29, 15)	+3 (-31, 18)	0.57
SCD	+18 (-49, 109)	-62 (-74, 4)	0.08
CNR1	-25 (-70, 70)	-4 (-66, 101)	0.80
CNR2	-30 (-83, 96)	-9 (-73, 111)	0.94

^a^ Significant within-group changes indicated: **P < 0.01.

Total number of individuals analysed varies slightly due to technical reasons.

Only percent changes are reported since gene expression is quantified in arbitrary units, absolute quantification is not performed.

11βHSD1: 11β-hydroxysteroid dehydrogenase type 1; CCL2: monocyte chemoattractant protein 1 (MCP-1); CNR1: cannabinoid receptor 1; CNR2: cannabinoid receptor 2; IL-6: interleukin 6; IQR: interquartile range; LPL: lipoprotein lipase; TNFα: tumour necrosis factor α; PPARγ: peroxisome proliferator activated receptor gamma; SCD: stearoyl CoA desaturase.

### Prediction of changes in leptin and adipose tissue linoleic acid by changes in physical activity, anthropometric measures and HbA1c

Markers of adipose tissue metabolism that responded significantly differently between the control and intervention groups, namely changes in serum leptin concentrations, adipose tissue linoleic acid content, and adipose tissue leptin gene expression levels, were analysed with linear regression to investigate the predictive ability of changes in physical activity (exercise time), anthropometric measures (weight, BMI and body fat mass), and HbA1c (Table 4). Decreases in circulating leptin concentrations were predicted by decreases in weight, BMI and body fat mass, but the latter was not statistically significant in the intervention group. Changes in leptin gene expression levels within adipose tissue were not significantly predicted by any of the selected parameters in either group. The only variable that explained significant variation in the change in adipose tissue linoleic acid content was change in exercise time, in the intervention group alone.

**Table 4:** Linear regression analysis for changes in serum leptin, changes in adipose tissue linoleic acid content, and changes in adipose tissue leptin gene expression in relation to changes in selected variables in control and intervention groups

	**∆ Serum leptin**		**∆ Adipose tissue linoleic acid**		**∆ Adipose tissue leptin gene expression**	
	**β**	**P**	**β**	**P**	**β**	**P**
**Control group**						
∆ Exercise time	-0.09	0.61	-0.17	0.31	0.10	0.64
∆ Weight	0.63	<0.0001	0.12	0.43	-0.01	0.96
∆ BMI	0.66	<0.0001	0.10	0.54	0.00	0.99
∆ Body fat mass	0.63	<0.0001	0.08	0.59	0.00	0.98
∆ HbA1c	0.20	0.20	-0.15	0.33	-0.19	0.34
∆ Linoleic acid	0.14	0.39	--	--	-0.34	0.086
∆ Leptin mRNA	-0.03	0.90	--	--	--	--
**Intervention group**						
∆ Exercise time	0.40	0.07	0.39	0.047	-0.43	0.11
∆ Weight	0.59	0.002	0.00	0.99	0.13	0.62
∆ BMI	0.57	0.003	-0.01	0.94	0.17	0.51
∆ Body fat mass	0.29	0.17	-0.09	0.63	0.06	0.82
∆ HbA1c	-0.15	0.46	0.09	0.64	0.01	0.97
∆ Linoleic acid	0.30	0.15	--	--	-0.46	0.06
∆ Leptin mRNA	-0.32	0.27	--	--	--	--

The size of the control and intervention groups is: n = 43 and n = 30 for serum leptin and adipose tissue linoleic acid; and n = 30 and n = 21 for adipose tissue leptin gene expression respectively, but the number of individuals analysed varies slightly due to technical reasons.

## Discussion

In the present analysis, using a subset of 68 year-old overweight-to-obese men and women exposed to physical activity on prescription in a randomized intervention trial for 6 months [3], we observed a decrease in circulating leptin concentrations, despite an increase in leptin mRNA in adipose tissue. Moreover, there was a small increase in the adipose tissue content of the w6 polyunsaturated fatty acid linoleic acid (18:2w6), even though no dietary recommendations were given. The original study showed improvements in metabolic and anthropometric measurements after the physical activity intervention. The current objective was to evaluate if such improvements could be related to changes in subcutaneous adipose tissue metabolism, as estimated by fatty acid composition, expression of genes encoding key proteins, and circulating adiponectin and leptin concentrations.

The data presented here for anthropometric and plasma parameters in the subset of individuals from whom adipose tissue biopsies were available are in line with our previously published data from the whole cohort [3]. While there were a number of improvements in metabolic parameters within both the control and intervention groups, the intervention group demonstrated more favourable changes in exercise time, weight, BMI, body fat mass and HbA1c compared to the control subjects, despite the fact that the control group had greater BMI, waist circumference, body fat mass, insulin and HOMA index at baseline. Here we show that circulating leptin concentrations decreased, but adiponectin concentrations were unchanged over the 6-month period of the intervention. We also report that the intervention group performed greater dietary improvements than the controls, but the extent of the dietary data was not sufficient for detailed investigation of nutrient composition.

While the fatty acid composition of adipose tissue reflects that of the diet over the past months to years [23], it is also modifiable by other factors since preferential uptake and release of certain fatty acids has been documented [24]. Obesity has been associated with a greater saturated fatty acid content of adipose tissue [17], and estimates of the activity of SCD within adipose tissue are increased in obesity and insulin resistance [19,20]. A study of obese subjects identified positive correlations between the w6-polyunsaturated fatty acid content of adipose tissue and measures of obesity in three different adipose tissue depots [25], while on the other hand, a 4-month marathon-training programme resulted in significant increases in the linoleic acid (18:2w6) content of subcutaneous adipose tissue in healthy men [26]. In the present study we find that 6 months of exercise on prescription resulted in a greater increase in the total w6-polyunsaturated fatty acid content of adipose tissue compared to the control group, and that linoleic acid largely accounted for this change. Since linoleic acid is preferentially retained in adipose tissue of both rats and rabbits when fatty acid mobilisation from adipose tissue is stimulated [27,28], an increase in physical activity might be expected to have similar effects. Indeed, changes in the adipose tissue linoleic acid content were not predicted by changes in weight, BMI, body fat mass or HbA1c, but the change in exercise time was a significant predictor in the intervention group, possibly implicating that the effect of increased physical activity on the linoleic acid content of adipose tissue was direct and not mediated via changes in adipose tissue mass or glucose control. However, changes in diet could also underlie the observation since greater dietary improvements were performed by individuals in the intervention group. However, although the dietary data were not detailed enough to permit investigation of dietary fat composition, analysis of reported changes in major fat sources suggested that no apparent alterations in the intake of w6-polyunsaturated fatty acids had occurred over the intervention period. There is an ongoing debate as to whether w6-polyunsaturated fatty acids are pro- or anti-inflammatory [29], but concentrations of linoleic acid in circulating cholesteryl esters were negatively correlated to plasma concentrations of CRP [30]. Therefore the increase in adipose tissue linoleic acid observed in the present study could be interpreted as a beneficial change, although the magnitude of the increase is very small (0.2 percentage points) and the biological relevance of such a change is unknown.

The expression levels in adipose tissue of genes encoding a number of proteins with important roles in adipose tissue metabolism were quantified. CCL2, IL-6, TNFα and CD68 were selected as markers of inflammation and macrophage infiltration, which are features of insulin resistant adipose tissue [14]. LPL and CD36 regulate lipid influx via hydrolysis of circulating triacylglycerols and fatty acid uptake respectively [31], while PPARγ is a key regulator of adipogenesis and adipocyte function [32]. Of the many adipokines produced and secreted by adipose tissue, leptin and adiponectin are two of the most important and are adipocyte-specific. Leptin regulates intake and expenditure of energy, while adiponectin increases insulin sensitivity and is anti-inflammatory [12]. The actions of the enzymes 11βHSD1 and SCD in adipose tissue have both been linked to insulin resistance [19,33]. Endocannabinoids increase food intake and weight gain and decrease energy expenditure via activation of the cannabinoid receptors CNR1 and CNR2 [34]. Activation of these receptors in the periphery (including adipose tissue) plays an important role in mediating the metabolic changes associated with obesity and insulin resistance [34]. Of these genes, changes in only the expression of leptin differed significantly between the control and intervention groups, with a median increase of 107% in the intervention group, compared to a median decrease of 20% in the control group. This result is unexpected given the concomitant decreases in circulating leptin concentrations, median changes of −6% in controls versus −13% in the intervention group (*P* = 0.01 for group comparison), and in light of the greater decrease in body weight and fat mass in the intervention group. Reports of decreased leptin concentrations in response to weight loss are widespread [35,36] and prior reports have shown decreased adipose tissue leptin expression and circulating leptin concentrations in response to exercise, although some contradictory results have also been reported [37,38]. The opposing changes observed for leptin mRNA expression in adipose tissue (increased) and for circulating leptin concentrations (decreased), at least in the intervention group, suggest that leptin mRNA levels are a poor marker of circulating leptin, implicating post transcriptional regulation of the leptin gene, as has been previously demonstrated in rats [39]. Indeed, changes in leptin gene expression and changes in circulating leptin concentrations were essentially unrelated to one other in the present study, which underlines the caution that should be employed when interpreting gene expression data and the importance of determining protein concentrations. The fact that the changes in circulating leptin concentrations were significantly predicted by changes in anthropometric measures, but not by changes in exercise time, might suggest that the lowering of leptin was mediated via decreases in fat mass, rather than by a direct effect of increased physical activity. Changes in HbA1c and the adipose tissue linoleic acid content were similarly unable to predict changes in circulating leptin.

Similar to the results presented here, a relative absence of changes in adipose tissue gene expression in response to physical activity (12-weeks of 3 supervised aerobic exercise sessions/week) was reported in obese individuals (BMI 33.3 kg/m^2^ at start, mean weight loss of 3.5 kg) [40]. Only expression of adiponectin increased, while IL-6, TNFα, CCL2, CCL3, leptin, CD68 and CD14 were all unchanged [40]. However, significant decreases in expression levels of IL-6, IL-8, TNFα CD68 and CD14 and an increase in adiponectin mRNA in adipose tissue of severely obese individuals who underwent major weight loss as a result of a 15-week lifestyle intervention consisting of hypocaloric diet and at least 2–3 hours of moderate-intensity physical activity 5 days/week (BMI 45.8 kg/m^2^ at start, mean weight loss of 18 kg) were reported [41]. This suggests that substantial changes in adipose tissue mass may be required in order to result in changes at the gene expression level.

These data suggest that the lifestyle modifications implemented, although sufficient to lead to reductions in fat mass (−0.5 kg in the controls and −1.5 kg in the intervention group) and circulating leptin concentrations, were not associated with alterations in circulating adiponectin. This observation is in line with a recent review that concluded that mild weight loss lowers leptin concentrations, but has no clear impact on adiponectin [35]. The clinical relevance of the increased adipose tissue content of linoleic acid is not clear. The relatively minor changes in adipose tissue metabolism achieved in the current study might reflect the fact that changes in adipose tissue mass were only small, or that changes in metabolism were too subtle to be detected by the techniques employed (primarily fatty acid composition and gene expression analysis). However, given that some changes in circulating parameters were documented (a greater improvement in HbA1c in the intervention compared to the control group), it may well be that modulation of adipose tissue metabolism was not directly responsible for such changes. The increased physical activity could have lead to alterations in other tissues, such as skeletal muscle, thereby mediating systemic improvements [42], but this was not investigated in the present study. Furthermore, the changes in diet (which were observed in both control and intervention groups, but greater in the latter) may have had effects on metabolism independent of those induced by an increased physical activity, but again, this remains unknown.

Strengths associated with the present study include the relatively large and homogenous study sample, as compared to previous studies in the field, and the implementation of a randomised controlled design. However, the cohort was not sufficiently large to address gender-differences. One limitation is that our data are derived from subcutaneous adipose tissue, rather than from the metabolically more active visceral depot. We cannot exclude the possibility that different adipose tissue depots within the body would respond differently to increases in physical activity. Furthermore, adipose tissue biopsies contain a mixture of cell types, although adipocytes predominate, and the cell sources of the mRNA of the genes investigated in this study is unknown. Finally, improvements in physical activity were also seen in the control group, which, combined with the greater dietary improvements in the intervention group, makes it harder to identify changes in metabolic parameters in the intervention group attributable to increased physical activity.

In summary, our results show that changes occurred in adipose tissue, as documented by decreased circulating leptin concentrations (despite increased leptin gene expression) and increased linoleic acid content in subcutaneous adipose tissue, after a 6-month period of lifestyle modification, primarily increased physical activity, in overweight-to-obese elderly individuals. However, these changes were relatively modest and we conclude that improvements in metabolic parameters induced by only a moderate increase in physical activity are unlikely to be driven solely by these changes in adipose tissue metabolism.

## Abbreviations

11βHSD1: 11β-hydroxysteroid dehydrogenase type 1; apo: Apolipoprotein; CCL2: Monocyte chemoattractant protein 1 (MCP-1); CNR1: Cannabinoid receptor 1; CNR2: Cannabinoid receptor 2; CRP: C-reactive protein; HbA1c: Glycosylated haemoglobin; HOMA: Homeostasis model assessment; IL-6: Interleukin 6; IQR: Interquartile range; LPL: Lipoprotein lipase; TNFα: Tumour necrosis factor α; PPARγ: Peroxisome proliferator activated receptor gamma; RPLP0: Ribosomal protein large P0; SCD: Stearoyl CoA desaturase.

## Competing interests

The authors declare that they have no competing interests.

## Authors' contributions

PS participated in study design, statistical analysis, data interpretation and manuscript writing. JS-J participated in study design, data collection and manuscript writing. LVK participated in study design and data collection. TC, MK, MH and KB participated in data collection. UdF participated in study design. M-LH conceived the study, participated in study design, data collection, data interpretation and manuscript writing. RMF participated in study design, statistical analysis, data interpretation and manuscript writing. All authors read and approved the final manuscript.

## Supplementary Material

Additional file 1 **Table S1.** Baseline characteristics and follow-up changes of selected variables in only those individuals from whom adipose gene expression data were available. Click here for file
