# Supplementary material for: Functional changes in adipose tissue in a randomised controlled trial of physical activity
Source: Lipids Health Dis. 2012 Jun 21;11:80. doi: 10.1186/1476-511X-11-80 (PMC3475078; doi:10.1186/1476-511X-11-80)
Supplement: Additional file 1 — Table S1. Baseline characteristics and follow-up changes of selected variables in only those individuals from whom adipose gene expression data were available. [file 1476-511X-11-80-S1.doc]

**Items to include when reporting a randomized trial in a journal or conference abstract**

| **Item** | **Description** | **Reported on line number** |
| --- | --- | --- |
| Title | Identification of the study as randomized | p1, lines 1-2 |
| Authors * | Contact details for the corresponding author |  |
| Trial design | Description of the trial design (e.g. parallel, cluster, non-inferiority) | p6, line 9-10 |
| Methods |  |  |
| Participants | Eligibility criteria for participants and the settings where the data were collected | p6, lines 3-9 |
| Interventions | Interventions intended for each group | p7, lines 5-9 |
| Objective | Specific objective or hypothesis | p5, lines 15-19 |
| Outcome | Clearly defined primary outcome for this report | p6, lines 23-25 |
| Randomization | How participants were allocated to interventions | p6, lines 11-12 |
| Blinding (masking) | Whether or not participants, care givers, and those assessing the outcomes were blinded to group assignment | p6, lines 12-14 |
| Results |  |  |
| Numbers randomized | Number of participants randomized to each group | p6, line 10 |
| Recruitment | Trial status | p6, line 8 |
| Numbers analysed | Number of participants analysed in each group | p6, lines 14 + 19 |
| Outcome | For the primary outcome, a result for each group and the estimated effect size and its precision | Tables 1-3 |
| Harms | Important adverse events or side effects | p7, line 10 |
| Conclusions | General interpretation of the results | p13, lines 13-18 |
| Trial registration | Registration number and name of trial register | Study initiated and approved by the local ethics committee in 2004, i.e. before 1 July 2005 from when all RCTs are required to be registered |
| Funding | Source of funding | p17, lines 11-17 |

**this item is specific to conference abstracts*
